# A novel dynamic nomogram based on contrast-enhanced computed tomography radiomics for prediction of glypican-3-positive hepatocellular carcinoma

**DOI:** 10.3389/fonc.2025.1640697

**Published:** 2025-10-15

**Authors:** Chunlong Zhao, Zheyu Zhou, Jiarun Zhang, Shuya Cao, Jiawei Xu, Cheng Wang, Jun Chen, Xiaoliang Xu, Chaobo Chen, Bing Han

**Affiliations:** ^1^ Department of General Surgery, Xishan People’s Hospital of Wuxi City, Wuxi, China; ^2^ Department of Hepatobiliary and Transplantation Surgery, The Affiliated Drum Tower Hospital of Nanjing University Medical School, Nanjing, China; ^3^ Department of General Surgery, Nanjing Drum Tower Hospital, Chinese Academy of Medical Sciences & Peking Union Medical College, Graduate School of Peking Union Medical College, Nanjing, China; ^4^ School of Medicine, Jiangsu University, Zhenjiang, China; ^5^ Hepatobiliary Center, The First Affiliated Hospital of Nanjing Medical University; Key Laboratory of Liver Transplantation, Chinese Academy of Medical Sciences; NHC Key Laboratory of Hepatobiliary Cancers, Nanjing, China; ^6^ Department of Pathology, The Affiliated Drum Tower Hospital of Nanjing University Medical School, Nanjing, China; ^7^ Department of General Surgery, The First Affiliated Hospital of Anhui Medical University, Hefei, China

**Keywords:** hepatocellular carcinoma, glypican-3, radiomics, computed tomography, prediction

## Abstract

**Background:**

The 5-year overall survival of hepatocellular carcinoma (HCC) is still poor. Since glypican-3 (GPC3) is highly expressed in most HCC but not in healthy or non-malignant livers, it may become an ideal therapeutic target for HCC. Thus, this study aimed to construct a dynamic nomogram based on contrast-enhanced computed tomography (CT) radiomics for predicting GPC3 expression.

**Methods:**

The medical data of consecutive HCC patients from Nanjing Drum Tower Hospital (from January 2020 to August 2023) were retrospectively reviewed. Based on the immunohistochemistry analysis, GPC3-positive was defined as a positive cell rate ≥ 10% (2+ and 3+). The 3D Slicer software and PyRadiomics were used to extract radiomics features on the arterial phase (AP) and venous phase (VP). A radiomics score (Radscore) was constructed using the most predictive features identified by the least absolute shrinkage and selection operator (LASSO) regression analysis. Univariate and multivariate analyses were performed to screen clinical risk factors associated with GPC3-positive. Finally, the Radscore and clinical risk factors were incorporated using logistic regression classification to construct a nomogram.

**Results:**

181 HCC patients were included according to the inclusion criteria. Among them, 106 were GPC3-positive, and 75 were GPC3-negative. Five radiomics features were finally screened, including three AP and two VP features. The nomogram model combining clinical risk factors (alpha-fetoprotein [AFP] ≥ 10 ng/mL, hepatitis B virus surface antigen [HBsAg]-negative, and age) and the Radscore (area under the receiver operating characteristic curve [AUROC] = 0.794) was superior to the clinical (AUROC = 0.724) and radiomics models (AUROC = 0.722), with good consistency in the calibration curve. The decision curve analysis (DCA) demonstrated that the nomogram had the highest net benefit for predicting GPC3-positive. The dynamic nomogram is freely available as a mobile application at https://zheyuzhou.shinyapps.io/GPC3nomogram/.

**Conclusions:**

Since the intra-tumor heterogeneity of HCC and potential complications brought by liver biopsy, our clinical prediction tool identified GPC3 status satisfactorily and might be helpful in clinical decision-making.

## Introduction

Hepatocellular carcinoma (HCC) is the most common primary liver cancer, with a poor 5-year overall survival, and its morbidity and mortality are still on the rise (1). Surgical resection is the optimal treatment of choice for HCC, but less than 30% of patients are suitable for radical procedures at first diagnosis (2). Although systemic therapies, including molecular targeted therapy, immunotherapy, and chemotherapy, have improved the median survival of intermediate and advanced HCC patients to about 20 months, the majority of patients still fail to achieve objective remission (3, 4). Thus, new therapeutic targets are urgently needed to improve the prognosis of intermediate and advanced HCC patients.

Glypican-3 (GPC3) is a cell membrane glycoprotein that is specifically expressed in liver, lung, and kidney tissues during fetal life but not in most adult tissues (5). Besides, GPC3 is highly expressed in HCC tissues, whereas it is under-expressed or not expressed in benign liver diseases (such as liver cirrhosis and focal nodular hyperplasia) (6). Soluble GPC3 is likewise found at elevated levels in HCC patients and undetectable in patients with hepatitis or healthy patients (7). Therefore, GPC3 may be a novel serum diagnostic marker and therapeutic target for HCC. The current study confirmed that GPC3-positive patients undergoing hepatectomy had significantly lower 5-year survival rates than GPC3-negative patients, and its expression was an independent prognostic factor for overall survival (8). Meanwhile, Wang YL et al. certified that GPC3 mRNA overexpression was significantly associated with recurrence of HCC in patients who underwent liver transplantation (9). For patients with advanced HCC, elevated expression of GPC3 may diminish the clinical benefit of bevacizumab plus atezolizumab treatment (10). Microvascular invasion (MVI) is an important indicator in liver pathology, and positive expression of GPC3 could significantly increase the incidence of MVI in HCC (11). Overall, high expression of GPC3 is associated with poor prognosis and unfavorable treatment response in HCC.

Radiomics is a high-throughput method capable of extracting a large number of quantitative imaging features from conventional images to better reflect tumor heterogeneity for prediction and diagnosis (12). There are several previous studies based on magnetic resonance imaging (MRI) radiomics to predict GPC3 expression. For instance, Chong H et al. developed a Gadoxetate Disodium-enhanced MRI radiomics model, which included 10 features, and its area under the receiver operating characteristic curve (AUROC) for distinguishing GPC3 status in combination with clinical factors could reach 0.943 (13). Due to the development of image post-processing techniques, three-dimensional (3D) reconstruction of liver vasculature and tumor volume measurements based on contrast-enhanced computed tomography (CT) are now increasingly performed in clinical practice (14). Contrast-enhanced CT plays a vital role in the diagnosis and treatment of HCC, while the prediction of GPC3 expression based on CT radiomics has not been thoroughly investigated.

## Methods

### Study design and included patients

Consecutive HCC patients’ medical data from Nanjing Drum Tower Hospital (from Jan. 2020 to Aug. 2023) were retrospectively reviewed. Because of the nature of the retrospective case-control study and unidentifiable patient information, the requirement for written informed consent was waived by the institutional review board of Nanjing Drum Tower Hospital. The inclusion criteria of this study were as follows: (1) patients with a first diagnosis of HCC who underwent liver resection; and (2) availability of complete clinicopathological and imaging data. The exclusion criteria were: (1) patients with recurrent HCC; (2) absence of preoperative contrast-enhanced CT imaging; (3) receipt of preoperative systemic or loco-regional therapies; and (4) presence of other primary malignancies. Importantly, two authors (ZYZ and CBC) independently performed the patient selection process to ensure consistency.

### Data collection

Included HCC patients’ blood test data were obtained within one week before the liver resection, including hepatitis B virus surface antigen (HBsAg), alpha-fetoprotein (AFP), des-γ-carboxy prothrombin (DCP), blood routine, and liver and coagulation functions. Furthermore, five inflammatory and three liver fibrosis serum markers were included, and their corresponding calculation formulas were described in the previous article (15). The number and size of tumors and the presence of macrovascular invasion (tumors invaded hepatic or portal vein branches (16)) were judged based on the preoperative contrast-enhanced CT. All included variables are presented in **Table 1**.

**Table 1:** Comparison of clinicopathology characteristics among the groups.

Variables	GPC3-negative (n = 75)	GPC3-positive (n = 106)	*P*
Age, years	62.0 ± 10.5	58.1 ± 12.0	**.024**
Gender			**.097**
Male, n (%)	63 (84.0%)	78 (73.6%)	
Female, n (%)	12 (16.0%)	28 (26.4%)	
HBsAg			**.053**
Negative, n (%)	20 (26.7%)	43 (40.6%)	
Positive, n (%)	55 (73.3%)	63 (59.4%)	
AFP, ng/mL			**<.001**
< 10	41 (54.7%)	27 (25.5%)	
≥ 10	34 (45.3%)	79 (74.5%)	
DCP, mAU/mL			.230
< 40	19 (25.3%)	19 (17.9%)	
≥ 40	56 (74.7%)	87 (82.1%)	
NE, ×10^9^/L	3.2 ± 1.3	3.1 ± 1.3	.629
LYM, ×10^9^/L	1.4 ± 0.5	1.5 ± 0.6	.246
M, ×10^9^/L	0.4 ± 0.2	0.4 ± 0.2	.659
PLT, ×10^9^/L	158.0 ± 63.4	163.6 ± 68.4	.576
ALT, U/L	28.3 ± 15.8	29.4 ± 20.2	.679
AST, U/L	28.5 ± 12.5	32.8 ± 21.4	**.120**
GGT, U/L	68.1 ± 69.2	78.8 ± 103.2	.436
TB, μmol/L	13.6 ± 6.2	14.6 ± 12.7	.510
ALB, g/L	40.1 ± 2.8	40.2 ± 3.0	.775
CRP, mg/L	10.2 ± 23.1	10.4 ± 20.6	.954
PT, seconds	11.5 ± 0.8	11.6 ± 0.8	.456
GLR^†^	53.3 ± 59.1	62.4 ± 91.3	.456
PNI^†^	47.1 ± 4.2	47.7 ± 4.4	.373
ANRI^†^	10.6 ± 7.0	12.9 ± 11.9	**.141**
NLR^†^	2.5 ± 1.6	2.2 ± 1.0	**.135**
MLR^†^	0.3 ± 0.1	0.3 ± 0.1	**.174**
APRI^#^	0.6 ± 0.4	0.7 ± 0.7	.364
FIB-4^#^	2.6 ± 1.7	2.8 ± 2.4	.691
GPR^#^	0.5 ± 0.6	0.6 ± 1.1	.353
Tumor number^			.280
Solitary, n (%)	66 (88.0%)	87 (82.1%)	
Multiple, n (%)	9 (12.0%)	19 (17.9%)	
Tumor size, cm^			.476
≤ 5	42 (56.0%)	65 (61.3%)	
> 5	33 (44.0%)	41 (38.7%)	
Macrovascular invasion^			.423
Absent, n (%)	63 (84.0%)	84 (79.2%)	
Present, n (%)	12 (16.0%)	22 (20.8%)	

Continuous variables are presented as mean ± standard deviation (SD). ^†^Inflammatory markers. ^#^Serum liver fibrosis diagnostic models. ^Preoperative imaging results.

HBsAg, hepatitis B virus surface antigen; AFP, alpha fetoprotein; DCP, des-γ-carboxy prothrombin; NE, neutrophil; LYM, lymphocyte; M, monocyte; PLT, platelet; ALT, alanine aminotransferase; AST, aspartate aminotransferase; GGT, γ-glutamyl transferase; TB, total bilirubin; ALB, albumin; CRP, c-reactive protein; PT, prothrombin time; GLR, γ-glutamyl transferase to lymphocyte ratio; PNI, prognostic nutritional index; ANRI, aspartate aminotransferase to neutrophil ratio index; NLR, neutrophil to lymphocyte ratio; MLR, monocyte to lymphocyte ratio; APRI, aspartate transaminase to platelet ratio index; FIB-4, fibrosis-4; GPR, γ-glutamyl transferase to platelet ratio. Bold values indicate variables with P-values less than 0.2.

### Contrast-enhanced CT scanning protocol

All patients underwent contrast-enhanced CT of the abdomen within two weeks prior to the liver resection. The contrast agent used was iohexol injection (35g, 100mL/COP bottle; GE Healthcare Shanghai Co. Ltd). CT was performed in the axial plane with 1.25-mm-thick sections using a 256-section (GE Revolution; GE Healthcare) multi-detector CT scanner. Patients were injected with 1.5mL/kg of iohexol after a routine unenhanced scan. Arterial phase (AP) images were acquired 30 seconds after injecting the contrast agent, and venous phase (VP) images began 30 seconds after the AP.

### Radiomics analysis

**Figure 1:**
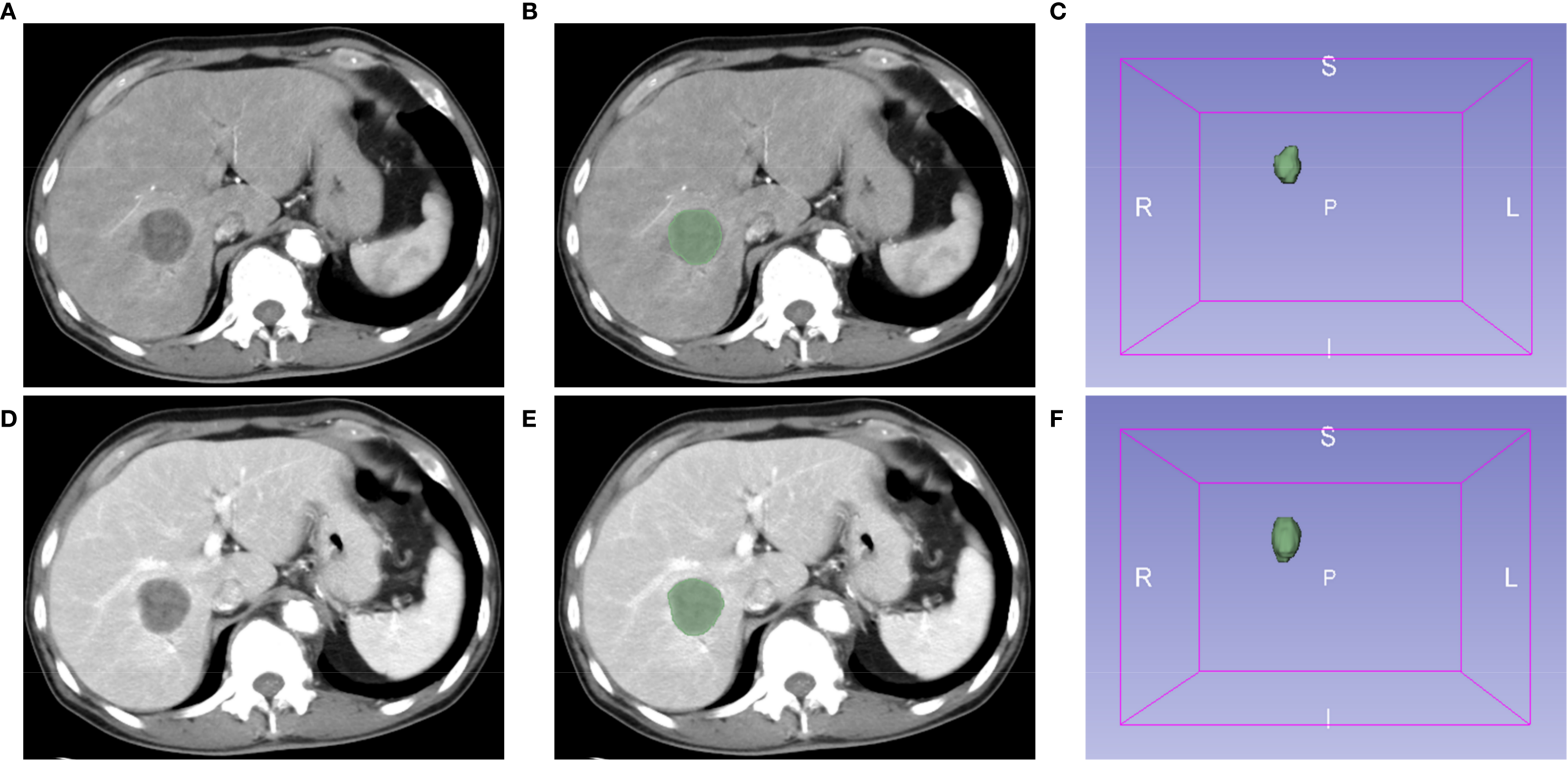
Extraction of radiomics features using the 3D Slicer software. Representative images of contrast-enhanced computed tomography (CT) arterial **(A)** and venous **(D)** phases. **(B, E)** Regions of interest (ROI). **(C, F)** The 3D reconstruction of tumors.

Images of contrast-enhanced CT arterial and venous phases of included patients were exported in the DICOM format. Two authors (CLZ and XLX) manually segmented tumors and outlined regions of interest (ROI) on each layer of images using the Segment Editor module of 3D Slicer software (version 5.4.0) (**Figure 1**). Images were resampled into voxels of 1×1×1 mm^3^ size using the SimpleITK module (version 2.3.1) in Python (version 3.9.12) to standardize voxel spacing. By default, B-Spline interpolation (order = 3) was applied for image resampling, while Nearest-Neighbor interpolation (order = 1) was used for ROI masks to preserve segmentation boundaries. The Python-based PyWavelets (version 1.3.0) package was used to perform wavelet transforms on all contrast-enhanced CT sequences to reduce image noise and normalize intensities. Finally, the PyRadiomics (version 3.1.0) package was used to extract 1,316 radiomics features from seven image types for each ROI, including shape features, first-order intensity features, and higher-order texture features derived from available filters (e.g., wavelet, Laplacian of Gaussian [LoG], and square) (17).

All 1,316 radiomics features extracted above were normalized using the z-score before filtering features. Subsequently, the intraclass correlation coefficients (ICCs) of the ROI features extracted by the two authors were calculated using the irr package (version 0.84.1) based on the R language (version 4.2.2), and features with coefficients > 0.8 were retained. According to the grouping of GPC3-positive and -negative, the most predictive features for GPC3 status were screened using the least absolute shrinkage and selection operator (LASSO) regression based on the glmnet package (version 4.1-8). Then, the optimal set of features was obtained at lambda.min using the 10-fold cross-validation. Eventually, the logistic regression model for predicting GPC3 status by radiomics features was built using the glm function of the R language, and the Radscore was calculated (18).

### Histopathological examination

The expression of GPC3 in HCC cells was evaluated using the criteria proposed by Takai H et al. under a light microscope (19). At least five randomly selected high-power fields within representative tumor areas were examined, and according to the proportion of positive HCC cells (brown reaction product present in the cell membrane and cytoplasm), expression grades were categorized into 0 to 3 +. Grade 0 corresponded to HCC cells with less than 5% positivity, and grade 1+ indicated 5-10% positivity. Grades 2+ and 3+ represented 10-50% and more than 50% positivity, respectively. Based on the above immunohistochemistry analysis, GPC3-positive was defined as a positive cell rate ≥ 10% (grades 2+ and 3+) (**Supplementary Figure S1**) (20). All liver resection specimens were independently analyzed by two pathologists, and any disagreements were resolved after discussion.

### Statistical analysis and model development

The χ^2^ and Mann-Whitney U tests were used to compare whether there were differences between the two groups for clinical variables. The subsequent multivariate logistic regression analysis included variables with *p*<0.2 in the univariate analysis. Similar to radiomics model (Radscore) establishment methods, the glm function was used to establish clinical and combined models. With the aim of using the combined model more conveniently, the DynNom package was used to exploit a mobile online prediction tool. At last, ROC curves, decision curve analyses (DCA), and calibration curves were used to evaluate the diagnostic accuracy, provided net benefit, and calibration of three models, respectively. Since the entire dataset was utilized for model construction, 10-fold cross-validation was performed for internal validation (21).

## Results

### Enrolled patients and baseline information

**Figure 2:**
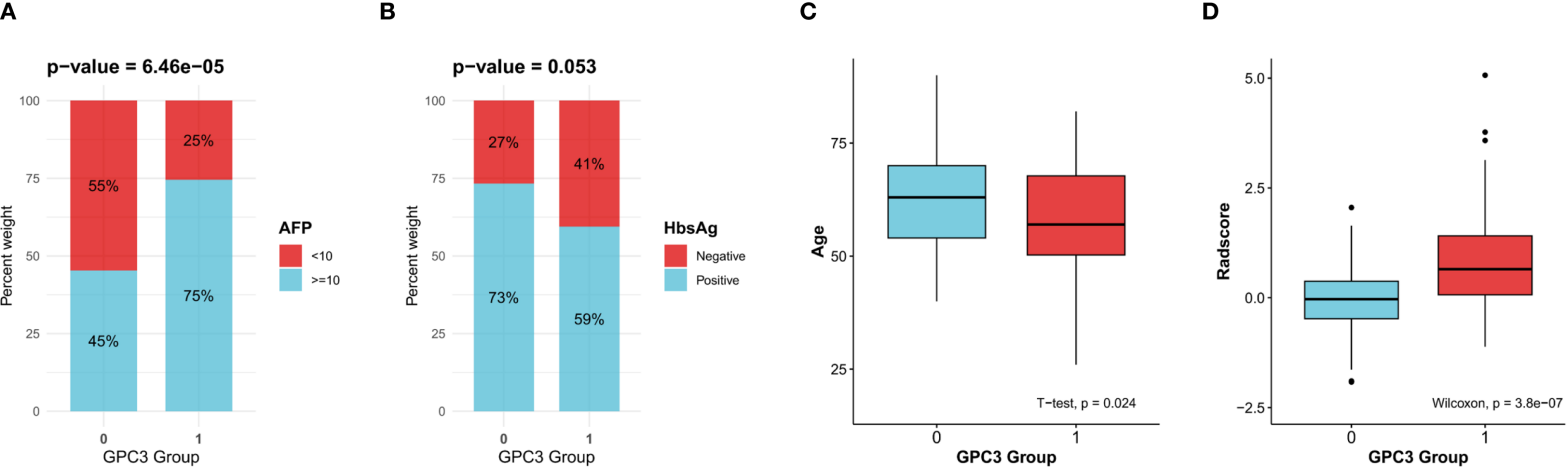
Clinical variables and the Radscore significantly associated with glypican-3 (GPC3)-positive expression. **(A)** Alpha-fetoprotein (AFP). **(B)** Hepatitis B virus surface antigen (HBsAg). **(C)** Age. **(D)** The radscore.

181 HCC patients from Nanjing Drum Tower Hospital who met the inclusion criteria were included in this retrospective case-control study. **Supplementary Figure S2** is the detailed flowchart of this study. Among them, 75 (41.4%) were GPC3-negative HCC, and 106 (58.6%) were GPC3-positive HCC. Then, the comparison of clinical variables was performed between the two groups, as shown in **Table 1**. AFP ≥ 10 ng/mL, age, HBsAg-negative, gender-female, AST, NLR, ANRI, and MLR were more correlated with GPC3 expression (*p*<0.2). Lastly, as presented in **Table 2**, the multivariate logistic regression identified four variables as independent predictors for GPC3-positive (*p*<0.05) (**Figure 2**).

**Table 2:** The multivariate analysis of GPC3-positive based on the univariate analysis.

Variables	OR (95% CI)	*P*
AFP (≥ 10 ng/mL)	2.95 (1.50-5.89)	**.002**
HBsAg (Negative)	0.33 (0.15-0.69)	**.004**
Age	0.96 (0.93-0.99)	**.015**
Gender (Male)	0.44 (0.17-1.04)	.067
AST	1.03 (0.99-1.06)	.137
ANRI	0.97 (0.91-1.03)	.321
NLR	0.85 (0.56-1.19)	.369
MLR	0.49 (0.01-22.63)	.709

GPC3, glypican-3; OR, odds ratio; CI, confidence interval; AFP, alpha fetoprotein; HBsAg, hepatitis B virus surface antigen; AST, aspartate aminotransferase; ANRI, aspartate aminotransferase to neutrophil ratio index; NLR, neutrophil to lymphocyte ratio; MLR, monocyte to lymphocyte ratio. Bold values indicate variables with P-values less than 0.05.

### Radiomics features analysis

**Figure 3:**
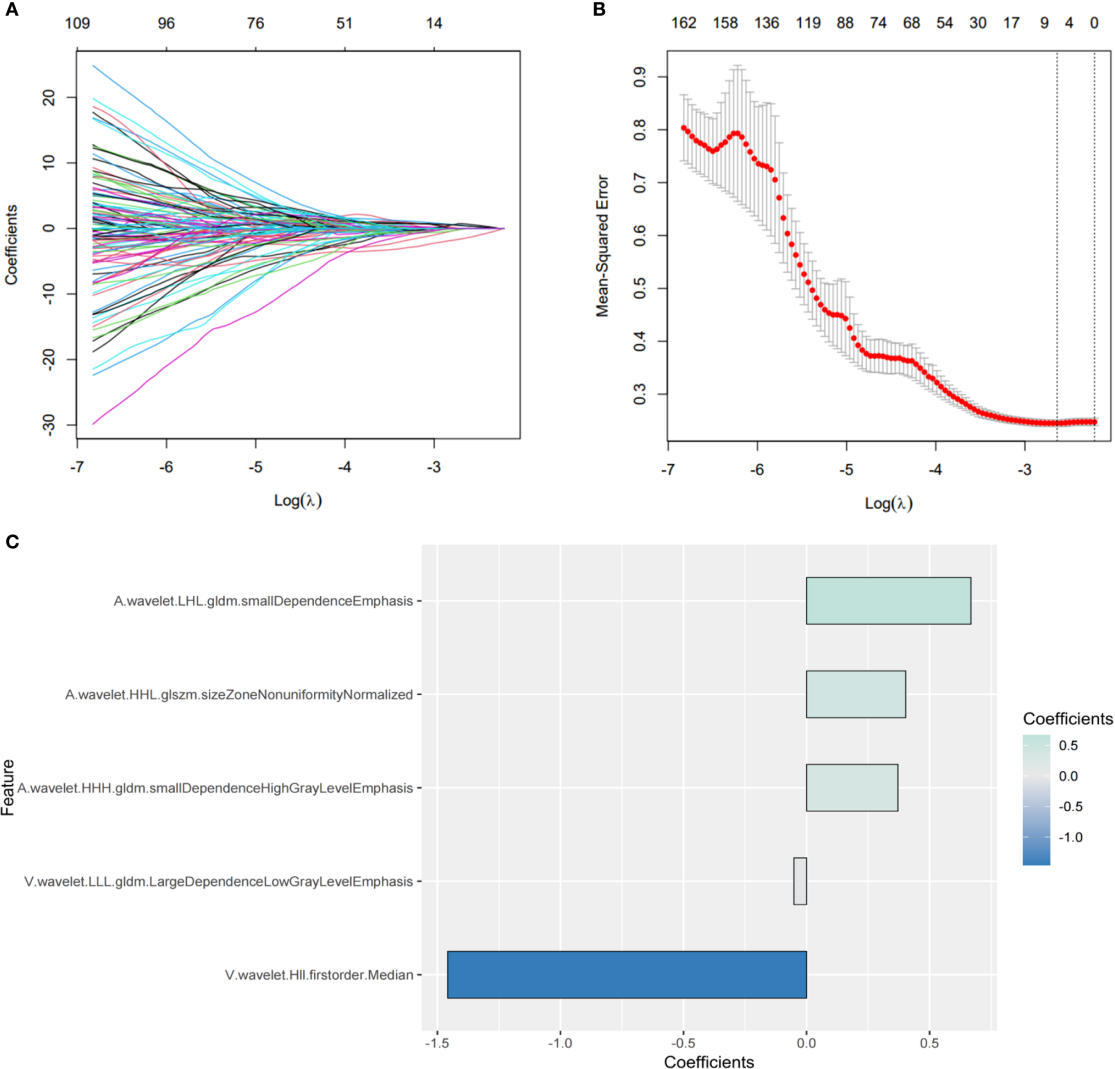
Screening of the most predictive radiomics features for glypican-3 (GPC3) status. **(A, B)** The process of the least absolute shrinkage and selection operator (LASSO) regression. **(C)** The coefficients of five crucial radiomics features.

746 features with ICCs > 0.8 were judged as stable features. The subsequent LASSO regression analysis (**Figures 3A, B**) finally identified five crucial radiomics features significantly related to GPC3 expression. The details and weighting coefficients of identified features were shown in **Figure 3C**. The calculation formula of the Radscore was as follows, the Radscore = 1.041 + 0.668 × (wavelet.LHL.gldm.SmallDependenceEmphasis) + 0.403 × (wavelet.HHL.glszm.SizeZoneNonUniformityNormalized) + 0.371 × (wavelet.HHH.gldm. SmallDependenceHighGrayLevelEmphasis) - 1.458 × (wavelet.HLL.firstorder.Median) - 0.051 × (wavelet.LLL.gldm.LargeDependenceLowGrayLevelEmphasis).

### Model development and evaluation

**Figure 4:**
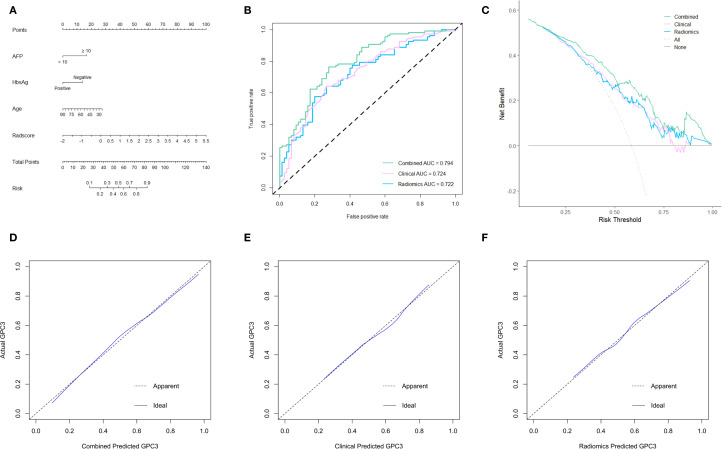
The establishment and evaluation of models. **(A)** The integrated nomogram incorporating clinical variables and the Radscore. Receiver operating characteristic (ROC) curves **(B)**, decision curve analyses (DCA) **(C)**, and calibration curves **(D-F)** of three established models.

The radiomics model consisted of five features in the Radscore, which had a sensitivity of 66.7%, a specificity of 76.4%, and an AUROC of 0.722. In contrast, the clinical model included three independent risk factors for GPC3-positive (AFP ≥ 10 ng/mL, HBsAg-negative, and age), which had a sensitivity of 62.7%, a specificity of 79.3%, and an AUROC of 0.724. To further improve the diagnostic accuracy of models, an integrated nomogram model incorporating the Radscore and clinical variables was established (**Figure 4A**). The dynamic nomogram is freely available as a mobile application at https://zheyuzhou.shinyapps.io/GPC3nomogram/ (the user interface is presented in **Supplementary Figure S3**). The integrated model improved the AUROC to 0.794 (**Figure 4B**) with a sensitivity of 85.9% and a specificity of 70.7%. Notably, the 95% confidence interval (CI) of the AUROC obtained using 10-fold cross-validation was 0.729-0.860 (**Supplementary Figure S4**). In addition, DCA showed that the integrated model could provide a higher net benefit than the radiomics model and the clinical model (**Figure 4C**). The calibration curves demonstrated close agreement between predicted GPC3 and actual GPC3 status (**Figures 4D-F**).

## Discussion and conclusion

The 5-year survival rate for advanced HCC patients is approximately 12% worldwide (22). At the same time, the 5-year cumulative recurrence rate for early and intermediate HCC patients treated with surgical resection, liver transplantation or ablation can be as high as 70% (23). Hence, exploring new therapeutic targets to improve the prognosis of advanced and recurrent patients is crucial to enhancing the overall long-term survival of HCC.

GPC3 has been proven to be a potential therapeutic target for HCC. Several phase I clinical trials have verified that chimeric antigen receptor (CAR)-GPC3-T cell therapy is safe for advanced HCC patients. Meanwhile, initially effective anti-tumor activity was observed (24, 25). Novel therapeutic strategies combining nanotechnology and CAR-T cell therapy are even providing new directions to enhance anti-tumor effects (26, 27). Moreover, it was worth noting that two previous phase II trials using GPC3-derived peptide vaccine as adjuvant therapy after radical procedures or radiofrequency ablation confirmed that the peptide vaccine significantly reduced recurrence of HCC, especially in GPC3-positive patients (28, 29). However, identifying target populations with high GPC3 expression is an essential issue in future clinical trials and clinical practice. In other words, GPC3-negative HCC patients cannot benefit from this targeted therapy.

Liver biopsy is the gold standard for assessing the nature and severity of liver diseases. A biopsy specimen measuring approximately 1/50000 of the total liver mass may not be representative of the entire liver parenchyma (30). On the other hand, the specimen’s length and diameter may affect the accuracy of the assessment (31). Clinically diagnosed intermediate to advanced HCC patients require liver biopsy for definitive pathological diagnosis prior to targeted therapy and immunotherapy. Since HCC has been proven to have extensive intra-tumor heterogeneity (32), the GPC3 expression status of biopsy specimens may be biased. Besides, advanced HCC is often combined with liver cirrhosis, which results in thrombocytopenia and coagulation disorders that increase the risk of biopsy-induced bleeding. The risk of needle tract implantation metastasis of HCC may also deprive some patients of conversion therapies (15). Therefore, radiomics, as an emerging noninvasive diagnostic method, has crucial advantages in predicting overall GPC3 expression in tumors. Although the radiomics features screened in this study (one first order feature and four grayscale texture features) were not present in previous HCC-related radiomics studies, SmallDependenceEmphasis was used to predict the infiltration status of CD8+ T cells prior to tumor treatment in head and neck squamous cell carcinomas (33), whereas SizeZoneNonUniformityNormalized was found to correlate with tumor grading (34). Moreover, Mukherjee S et al. reported that first order. Median was valuable in the early detection of pancreatic ductal adenocarcinoma (35). These suggested that CT-based radiomics could effectively reflect tumor heterogeneity.

In this study, age was found to be a protective factor for GPC3-positive, consistent with the findings of Zhang N et al. (20). The worse prognosis of GPC3-positive patients may be associated with higher tumor invasiveness in this subtype of HCC, manifested by more frequent vascular invasion, higher tumor number, and later tumor staging (36, 37). A basic study explored the potential mechanism of this phenomenon. HCC cells HepG2 expressing high levels of GPC3 had significant epithelial-mesenchymal transition-like alterations. Simultaneously, cell scratch and transwell assays confirmed that these cells had enhanced migration and invasion capabilities (37). Of note, a previous analysis based on 10,145 patients from the Surveillance Epidemiology and End Results (SEER) database showed that the later the tumor stage at diagnosing HCC, the younger the patient and the faster the HCC growth (38). Another large-sample retrospective study also reported that younger patients had more aggressive tumor factors (39). This might explain why younger HCC patients are more likely to express GPC3.

AFP is a traditional biomarker for HCC (40). AFP-secreting HCC is more aggressive than AFP-negative HCC, and single-cell sequencing revealed that AFP-positive HCC patients had a suppressive tumor immune microenvironment (41). Bevacizumab plus atezolizumab has emerged as a first-line systemic treatment option for advanced HCC patients. In-depth molecular analysis demonstrated that high AFP and GPC3 (oncofetal genes) expressions were associated with reduced clinical benefit (10). This confirmed that there might be a consistency in AFP and GPC3 expression in HCC patients with poor prognosis. Furthermore, initial and updated meta-analyses verified that combining elevated AFP and GPC3 could improve the accuracy of diagnosing HCC (42, 43). Since Morford LA et al. reported that AFP regulator 2 is also a regulator of GPC3 (44), this explains the regulatory mechanism why AFP-positive (≥ 10 ng/mL) is a risk factor for GPC3-positive.

A previous study reported that 5 features selected from the contrast-enhanced CT AP in combination with AFP could predict GPC3-positive expression. However, the authors did not provide a formula for the Radscore or construct a nomogram model (45). In other words, readers cannot refer to this result for clinical prediction. The development of a mobile application based on the user-friendly nomogram represents a major strength and a key novelty of this study. In addition, features were simultaneously extracted from both the AP and VP in our study, thereby fully utilizing the information provided by contrast-enhanced CT imaging. These help physicians’ clinical decision-making and provides a stronger foundation for predicting GPC3 expression in HCC patients based on contrast-enhanced CT radiomics.

The nature of a single-center retrospective study is a major limitation of this study, which limited the sample size and inevitably led to selection bias. For instance, previous studies reported that HBsAg-positive was associated with GPC3-positive. Gong L et al. reported that out of 80 GPC3-positive HCC patients, 62 (77.5%) were HBsAg-positive. In contrast, in 22 GPC3-negative patients, the percentage was only 31.8% (7/22) (46). Moreover, a study including 755 HCC patients showed that HBsAg-positive rates in GPC3-positive and -negative patients were 78.7% and 72.1%, respectively (*p*=0.042) (47). Since patients lacking preoperative contrast-enhanced CT and DCP were excluded from this study among consecutive patients, the resulting selection bias could be the reason why HBsAg-negative is a predictor of high GPC3 expression. However, consecutive patients were strictly screened based on the predefined criteria to minimize bias. Although the lack of an external validation cohort prevents the generalizability of the model from being fully verified, 10-fold cross-validation is a well acknowledged approach to improve the model stability. We presented comprehensive baseline data to allow comparison with other populations, but multi-center studies are still required to validate our findings in the future.

In conclusion, our clinical prediction tool identified GPC3 status satisfactorily and might be helpful in clinical decision-making as the intra-tumor heterogeneity of HCC and potential complications brought by liver biopsy. For surgeons, early identification of high-risk GPC3-positive patients (risk stratification) may assist in adopting a wider resection margin or anatomical hepatectomy. For oncologists, the proposed nomogram may help identify appropriate candidate populations for enrollment in future GPC3-related clinical trials. For pathologists, our model may serve as a reference to improve diagnostic accuracy.

## Data Availability

The raw data supporting the conclusions of this article will be made available by the authors, without undue reservation.

## References

[1] Benson AB D'Angelica MI Abbott DE Anaya DA Anders R Are C. Hepatobiliary cancers, version 2.2021, NCCN clinical practice guidelines in oncology. J Natl Compr Canc Netw. (2021) 19:541–65. doi: 10.6004/jnccn.2021.0022, PMID: 34030131

[2] Zhou J Sun H Wang Z Cong W Zeng M Zhou W. Guidelines for the diagnosis and treatment of primary liver cancer (2022 edition). Liver Cancer. (2023) 12:405–44. doi: 10.1159/000530495, PMID: 37901768 PMC10601883

[3] Finn RS Qin S Ikeda M Galle PR Ducreux M Kim TY. Atezolizumab plus bevacizumab in unresectable hepatocellular carcinoma. N Engl J Med. (2020) 382:1894–905. doi: 10.1056/NEJMoa1915745, PMID: 32402160

[4] Qin S Chan SL Gu S Bai Y Ren Z Lin X. Camrelizumab plus rivoceranib versus sorafenib as first-line therapy for unresectable hepatocellular carcinoma (CARES-310): a randomized, open-label, international phase 3 study. Lancet. (2023) 402:1133–46. doi: 10.1016/S0140-6736(23)00961-3, PMID: 37499670

[5] Schepers EJ Glaser K Zwolshen HM Hartman SJ Bondoc AJ. Structural and functional impact of posttranslational modification of glypican-3 on liver carcinogenesis. Cancer Res. (2023) 83:1933–40. doi: 10.1158/0008-5472.CAN-22-3895, PMID: 37027004 PMC10267680

[6] Zhu ZW Friess H Wang L Abou-Shady M Zimmermann A Lander AD. Enhanced glypican-3 expression differentiates the majority of hepatocellular carcinomas from benign hepatic disorders. Gut. (2001) 48:558–64. doi: 10.1136/gut.48.4.558, PMID: 11247902 PMC1728256

[7] Capurro M Wanless IR Sherman M Deboer G Shi W Miyoshi E. Glypican-3: a novel serum and histochemical marker for hepatocellular carcinoma. Gastroenterology. (2003) 125:89–97. doi: 10.1016/S0016-5085(03)00689-9, PMID: 12851874

[8] Shirakawa H Suzuki H Shimomura M Kojima M Gotohda N Takahashi S. Glypican-3 expression is correlated with poor prognosis in hepatocellular carcinoma. Cancer Sci. (2009) 100:1403–7. doi: 10.1111/j.1349-7006.2009.01206.x, PMID: 19496787 PMC11158276

[9] Wang YL Zhu ZJ Teng DH Yao Z Gao W Shen ZY. Glypican-3 expression and its relationship with recurrence of HCC after liver transplantation. World J Gastroenterol. (2012) 18:2408–14. doi: 10.3748/wjg.v18.i19.2408, PMID: 22654434 PMC3353377

[10] Zhu AX Abbas AR de Galarreta MR Guan Y Lu S Koeppen H. Molecular correlates of clinical response and resistance to atezolizumab in combination with bevacizumab in advanced hepatocellular carcinoma. Nat Med. (2022) 28:1599–611. doi: 10.1038/s41591-022-01868-2, PMID: 35739268

[11] Wang Z Cao L Wang J Wang H Ma T Yin Z. A novel predictive model of microvascular invasion in hepatocellular carcinoma based on differential protein expression. BMC Gastroenterol. (2023) 23:89. doi: 10.1186/s12876-023-02729-z, PMID: 36973651 PMC10041792

[12] Bo Z Song J He Q Chen B Chen Z Xie X. Application of artificial intelligence radiomics in the diagnosis, treatment, and prognosis of hepatocellular carcinoma. Comput Biol Med. (2024) 173:108337. doi: 10.1016/j.compbiomed.2024.108337, PMID: 38547656

[13] Chong H Gong Y Zhang Y Dai Y Sheng R Zeng M. Radiomics on gadoxetate disodium-enhanced MRI: non-invasively identifying glypican 3-positive hepatocellular carcinoma and postoperative recurrence. Acad Radiol. (2023) 30:49–63. doi: 10.1016/j.acra.2022.04.006, PMID: 35562264

[14] Cao S Zhou Z Chen C Li W Liu J Xu J. Early identification of hepatocellular carcinoma patients at high-risk of recurrence using the ADV score: a multicenter retrospective study. World J Surg Oncol. (2024) 22:240. doi: 10.1186/s12957-024-03523-1, PMID: 39244533 PMC11380786

[15] Zhou Z Chen C Sun M Xu X Liu Y Liu Q. A decision tree model to predict liver cirrhosis in hepatocellular carcinoma patients: a retrospective study. PeerJ. (2023) 11:e15950. doi: 10.7717/peerj.15950, PMID: 37641600 PMC10460570

[16] Costentin CE Ferrone CR Arellano RS Ganguli S Hong TS Zhu AX. Hepatocellular carcinoma with macrovascular invasion: defining the optimal treatment strategy. Liver Cancer. (2017) 6:360–74. doi: 10.1159/000481315, PMID: 29234639 PMC5704715

[17] van Griethuysen JJM Fedorov A Parmar C Hosny A Aucoin N Narayan V. Computational radiomics system to decode the radiographic phenotype. Cancer Res. (2017) 77:e104–7. doi: 10.1158/0008-5472.CAN-17-0339, PMID: 29092951 PMC5672828

[18] Wu S Zhan W Liu L Xie D Yao L Yao H. Pretreatment radiomic biomarker for immunotherapy responder prediction in stage IB-IV NSCLC (LCDigital-IO Study): a multicenter retrospective study. J Immunother Cancer. (2023) 11:e007369. doi: 10.1136/jitc-2023-007369, PMID: 37865396 PMC10603353

[19] Takai H Kato A Ishiguro T Kinoshita Y Karasawa Y Otani Y. Optimization of tissue processing for immunohistochemistry for the detection of human glypican-3. Acta Histochem. (2010) 112:240–50. doi: 10.1016/j.acthis.2008.11.025, PMID: 19246079

[20] Zhang N Wu M Zhou Y Yu C Shi D Wang C. Radiomics nomogram for prediction of glypican-3 positive hepatocellular carcinoma based on hepatobiliary phase imaging. Front Oncol. (2023) 13:1209814. doi: 10.3389/fonc.2023.1209814, PMID: 37841420 PMC10570799

[21] Steyerberg EW Harrell FE Jr Borsboom GJ Eijkemans MJ Vergouwe Y Habbema JD. Internal validation of predictive models: efficiency of some procedures for logistic regression analysis. J Clin Epidemiol. (2001) 54:774–81. doi: 10.1016/S0895-4356(01)00341-9, PMID: 11470385

[22] Krishnamurthy S Gilot D Ahn SB Lam V Shin JS Guillemin GJ. Involvement of kynurenine pathway in hepatocellular carcinoma. Cancers (Basel). (2021) 13:5180. doi: 10.3390/cancers13205180, PMID: 34680327 PMC8533819

[23] Nevola R Ruocco R Criscuolo L Villani A Alfano M Beccia D. Predictors of early and late hepatocellular carcinoma recurrence. World J Gastroenterol. (2023) 29:1243–60. doi: 10.3748/wjg.v29.i8.1243, PMID: 36925456 PMC10011963

[24] Pang N Shi J Qin L Chen A Tang Y Yang H. IL-7 and CCL19-secreting CAR-T cell therapy for tumors with positive glypican-3 or mesothelin. J Hematol Oncol. (2021) 14:118. doi: 10.1186/s13045-021-01128-9, PMID: 34325726 PMC8323212

[25] Shi D Shi Y Kaseb AO Qi X Zhang Y Chi J. Chimeric antigen receptor-glypican-3 T-cell therapy for advanced hepatocellular carcinoma: results of phase I trials. Clin Cancer Res. (2020) 26:3979–89. doi: 10.1158/1078-0432.CCR-19-3259, PMID: 32371538

[26] Yuan Y Sun W Xie J Zhang Z Luo J Han X. RNA nanotherapeutics for hepatocellular carcinoma treatment. Theranostics. (2025) 15:965–92. doi: 10.7150/thno.102964, PMID: 39776807 PMC11700867

[27] Ma W Zhu D Li J Chen X Xie W Jiang X. Coating biomimetic nanoparticles with chimeric antigen receptor T cell-membrane provides high specificity for hepatocellular carcinoma photothermal therapy treatment. Theranostics. (2020) 10:1281–95. doi: 10.7150/thno.40291, PMID: 31938065 PMC6956810

[28] Sawada Y Yoshikawa T Ofuji K Yoshimura M Tsuchiya N Takahashi M. Phase II study of the GPC3-derived peptide vaccine as an adjuvant therapy for hepatocellular carcinoma patients. Oncoimmunology. (2016) 5:e1129483. doi: 10.1080/2162402X.2015.1129483, PMID: 27467945 PMC4910752

[29] Taniguchi M Mizuno S Yoshikawa T Fujinami N Sugimoto M Kobayashi S. Peptide vaccine as an adjuvant therapy for glypican-3-positive hepatocellular carcinoma induces peptide-specific CTLs and improves long prognosis. Cancer Sci. (2020) 111:2747–59. doi: 10.1111/cas.14497, PMID: 32449239 PMC7419030

[30] Bravo AA Sheth SG Chopra S. Liver biopsy. N Engl J Med. (2001) 344:495–500. doi: 10.1056/NEJM200102153440706, PMID: 11172192

[31] Patel K Sebastiani G. Limitations of non-invasive tests for assessment of liver fibrosis. JHEP Rep. (2020) 2:100067. doi: 10.1016/j.jhepr.2020.100067, PMID: 32118201 PMC7047178

[32] Ma L Wang L Khatib SA Chang CW Heinrich S Dominguez DA. Single-cell atlas of tumor cell evolution in response to therapy in hepatocellular carcinoma and intrahepatic cholangiocarcinoma. J Hepatol. (2021) 75:1397–408. doi: 10.1016/j.jhep.2021.06.028, PMID: 34216724 PMC8604764

[33] Wang CY Ginat DT. Preliminary computed tomography radiomics model for predicting pretreatment CD8+ T-cell infiltration status for primary head and neck squamous cell carcinoma. J Comput Assist Tomogr. (2021) 45:629–36. doi: 10.1097/RCT.0000000000001149, PMID: 34519454

[34] Committeri U Fusco R Di Bernardo E Abbate V Salzano G Maglitto F. Radiomics metrics combined with clinical data in the surgical management of early-stage (cT1-T2 N0) tongue squamous cell carcinomas: A preliminary study. Biol (Basel). (2022) 11:468. doi: 10.3390/biology11030468, PMID: 35336841 PMC8945467

[35] Mukherjee S Patra A Khasawneh H Korfiatis P Rajamohan N Suman G. Radiomics-based machine-learning models can detect pancreatic cancer on prediagnostic computed tomography scans at a substantial lead time before clinical diagnosis. Gastroenterology. (2022) 163:1435–46.e3. doi: 10.1053/j.gastro.2022.06.066, PMID: 35788343 PMC12285712

[36] Fu SJ Qi CY Xiao WK Li SQ Peng BG Liang LJ. Glypican-3 is a potential prognostic biomarker for hepatocellular carcinoma after curative resection. Surgery. (2013) 154:536–44. doi: 10.1016/j.surg.2013.02.014, PMID: 23601901

[37] Wu Y Liu H Weng H Zhang X Li P Fan CL. Glypican-3 promotes epithelial-mesenchymal transition of hepatocellular carcinoma cells through ERK signaling pathway. Int J Oncol. (2015) 46:1275–85. doi: 10.3892/ijo.2015.2827, PMID: 25572615

[38] Gao TM Bai DS Qian JJ Zhang C Jin SJ Jiang GQ. The growth rate of hepatocellular carcinoma is different with different TNM stages at diagnosis. Hepatobiliary Pancreat Dis Int. (2021) 20:330–6. doi: 10.1016/j.hbpd.2021.02.005, PMID: 33637452

[39] Su CW Lei HJ Chau GY Hung HH Wu JC Hsia CY. The effect of age on the long-term prognosis of patients with hepatocellular carcinoma after resection surgery: a propensity score matching analysis. Arch Surg. (2012) 147:137–44. doi: 10.1001/archsurg.2011.288, PMID: 22006855

[40] Zhou Z Liu J Xu X. A commentary on ‘Prothrombin induced by vitamin K Absence-II versus alpha-fetoprotein in detection of both resectable hepatocellular carcinoma and early recurrence after curative liver resection: a retrospective cohort study’ (Int J Surg 2022;105:106843). Int J Surg. (2023) 109:3656–8. doi: 10.1097/JS9.0000000000000119, PMID: 36906781 PMC10651297

[41] He H Chen S Fan Z Dong Y Wang Y Li S. Multi-dimensional single-cell characterization revealed suppressive immune microenvironment in AFP-positive hepatocellular carcinoma. Cell Discov. (2023) 9:60. doi: 10.1038/s41421-023-00563-x, PMID: 37336873 PMC10279759

[42] Song H Wang J Zhang H Wu Y Wang K Wang X. Combination of serum alpha-fetoprotein, PIVKA-II; and glypican-3 in diagnosis of hepatocellular carcinoma: a meta-analysis. Zhejiang Da Xue Xue Bao Yi Xue Ban. (2024) 53:131–9. doi: 10.3724/zdxbyxb-2023-0483, PMID: 38310085 PMC10945496

[43] Xu D Su C Sun L Gao Y Li Y. Performance of serum glypican 3 in diagnosis of hepatocellular carcinoma: A meta-analysis. Ann Hepatol. (2019) 18:58–67. doi: 10.5604/01.3001.0012.7863, PMID: 31113610

[44] Morford LA Davis C Jin L Dobierzewska A Peterson ML Spear BT. The oncofetal gene glypican 3 is regulated in the postnatal liver by zinc fingers and homeoboxes 2 and in the regenerating liver by alpha-fetoprotein regulator 2. Hepatology. (2007) 46:1541–7. doi: 10.1002/hep.21825, PMID: 17668883

[45] Sun S Xiao S Jiang Z Xiao J He Q Wang M. Radiomic analysis of contrast-enhanced CT predicts glypican 3-positive hepatocellular carcinoma. Curr Med Imaging. (2024) 20:e15734056277475. doi: 10.2174/0115734056277475240215115629, PMID: 38462828

[46] Gong L Wei LX Ren P Zhang WD Liu XY Han XJ. Dysplastic nodules with glypican-3 positive immunostaining: a risk for early hepatocellular carcinoma. PLoS One. (2014) 9:e87120. doi: 10.1371/journal.pone.0087120, PMID: 24498024 PMC3909016

[47] Yan B Wei JJ Qian YM Zhao XL Zhang WW Xu AM. Expression and clinicopathologic significance of glypican 3 in hepatocellular carcinoma. Ann Diagn Pathol. (2011) 15:162–9. doi: 10.1016/j.anndiagpath.2010.10.004, PMID: 21371925

