# Supplementary material for: A novel dynamic nomogram based on contrast-enhanced computed tomography radiomics for prediction of glypican-3-positive hepatocellular carcinoma
Source: Front Oncol. 2025 Oct 15;15:1640697. doi: 10.3389/fonc.2025.1640697 (PMC12568392; doi:10.3389/fonc.2025.1640697)
Supplement: Supplementary file 1 [file DataSheet1.docx]

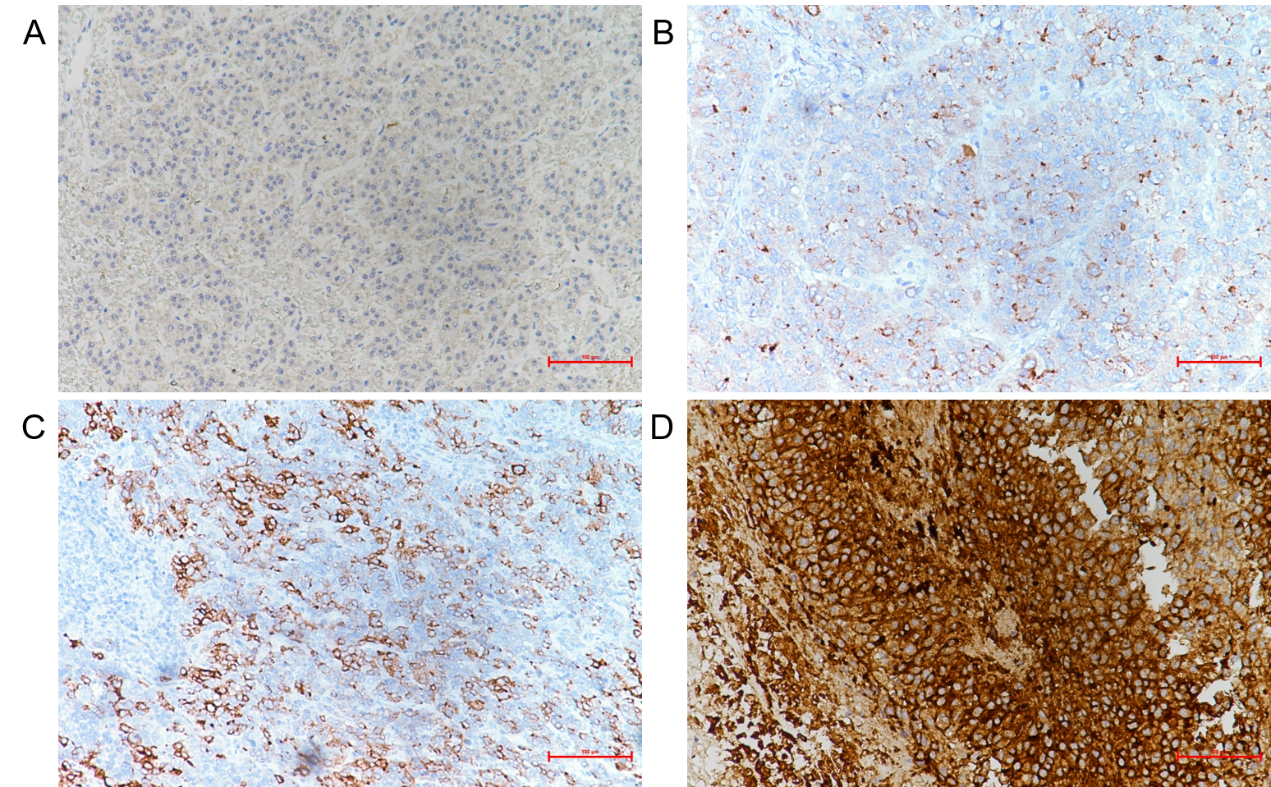


**Fig. S1** Typical pathological section images of glypican-3 (GPC3) expression. **(A and B)** GPC3-negative: grades – and +. **(C and D)** GPC3-positive: grades 2+ and 3+. Scale bar = 100μm.


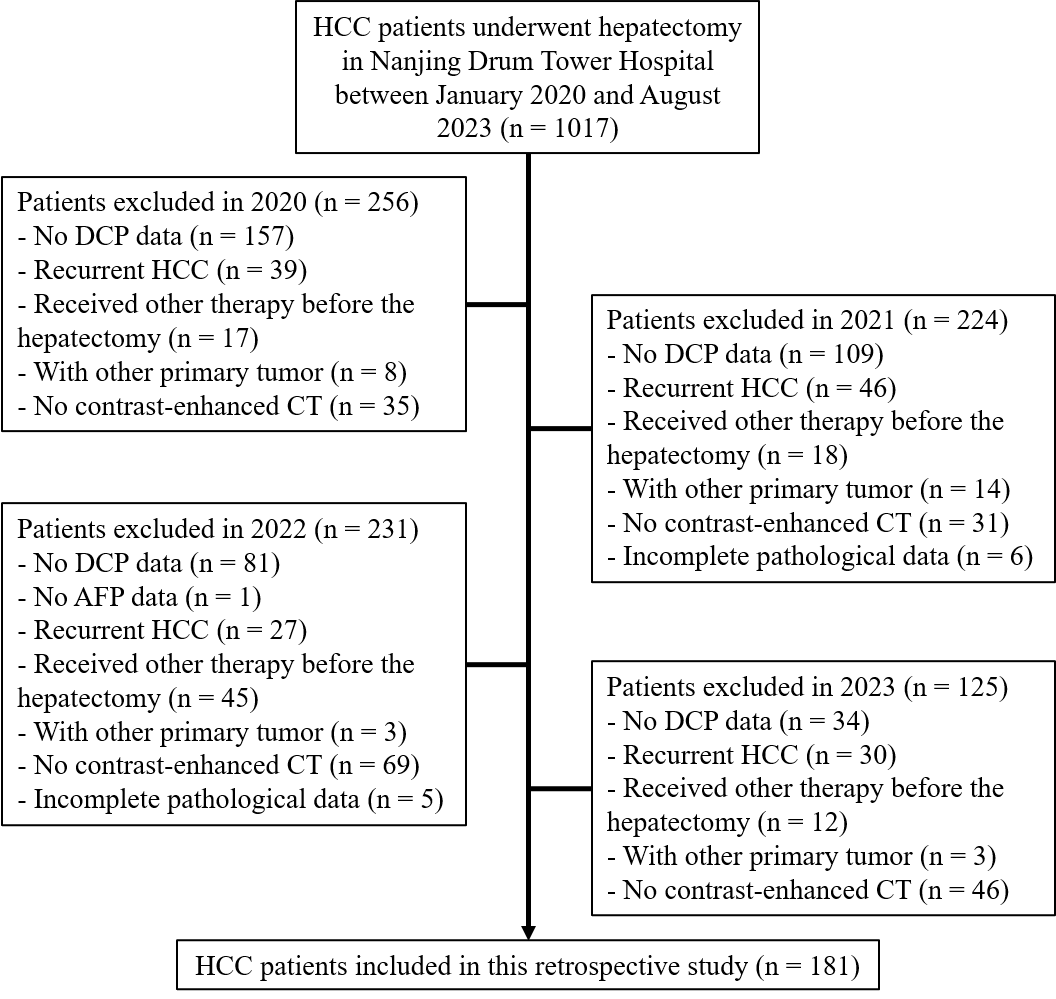


**Fig. S2** Patient selection flowchart. HCC, hepatocellular carcinoma; DCP, des-γ-carboxy prothrombin; CT, computed tomography; AFP, alpha-fetoprotein


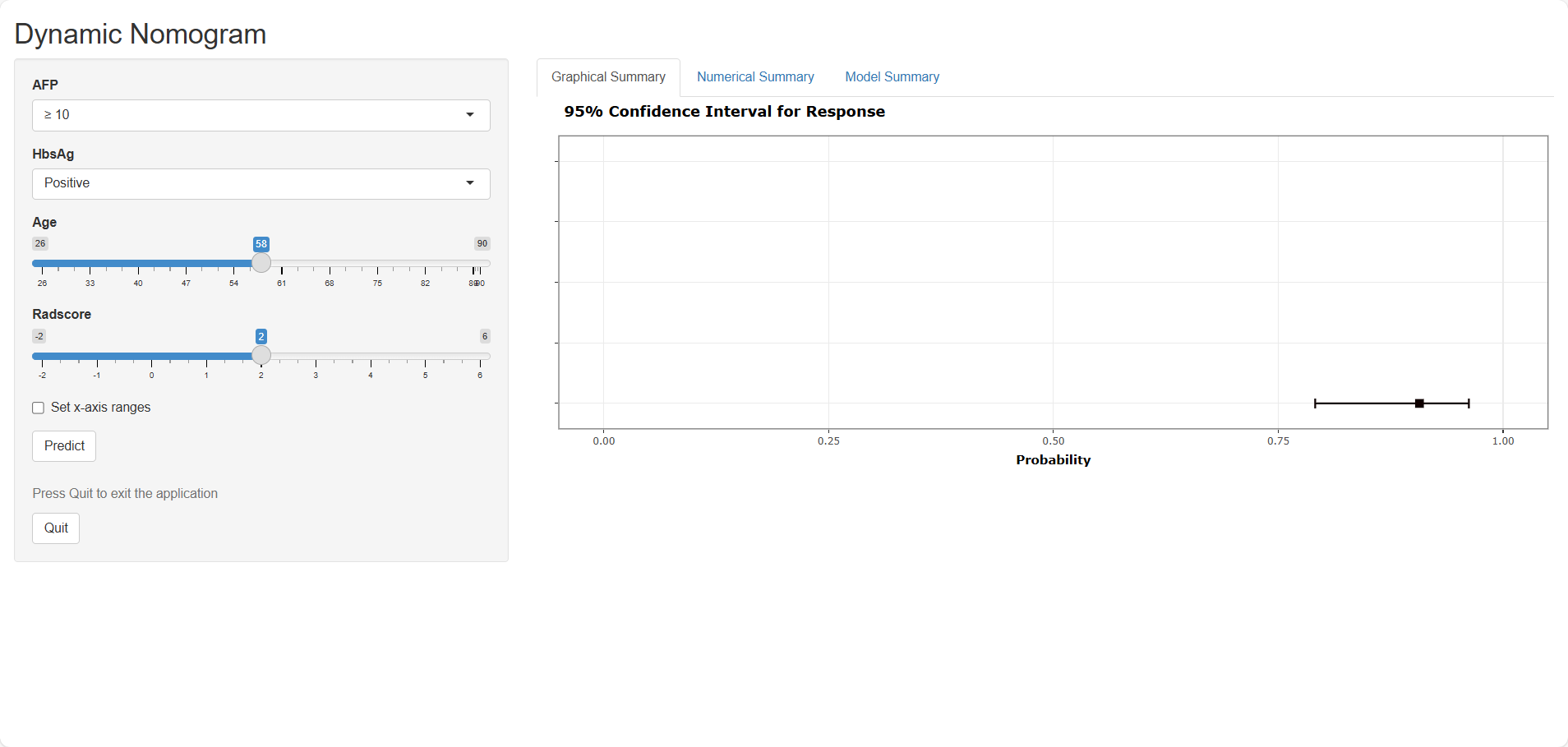


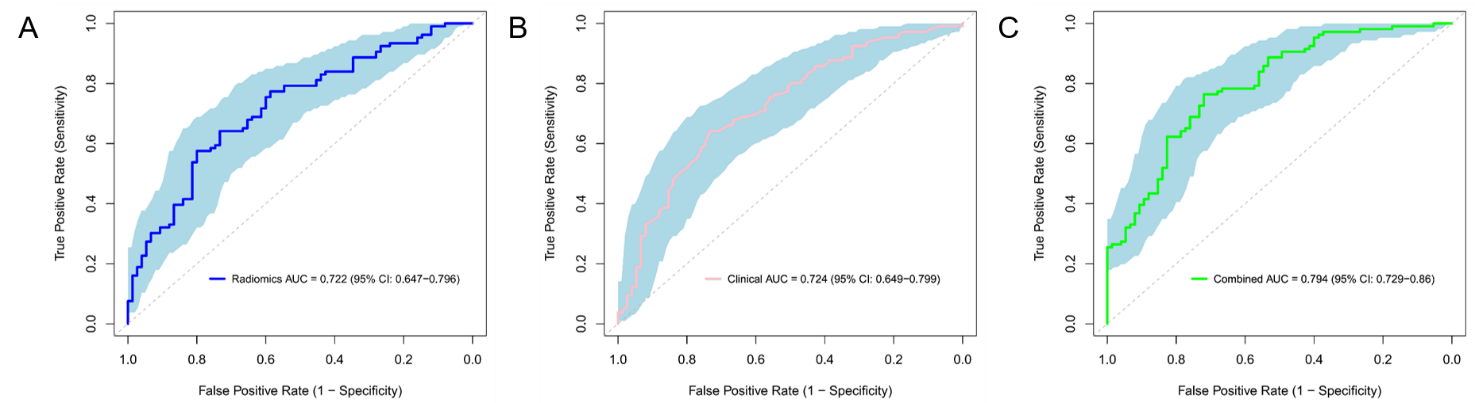
**Fig. S3** The user interface of the online prediction tool.

**Fig. S4** Receiver operating characteristic (ROC) curves of the constructed models using 10-fold cross-validation. **(A)** Radiomics model. **(B)** Clinical model. **(C)** Combined model. AUC, area under the curve; CI, confidence interval.
